# Supplementary figures and images for: Efficacy and safety of Weifuchun tablet for chronic atrophic gastritis: A systematic review and meta-analysis
Source: PLoS One. 2023 Apr 13;18(4):e0284411. doi: 10.1371/journal.pone.0284411 (PMC10101393; doi:10.1371/journal.pone.0284411)

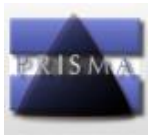

## PRISMA 2009 Flow Diagram

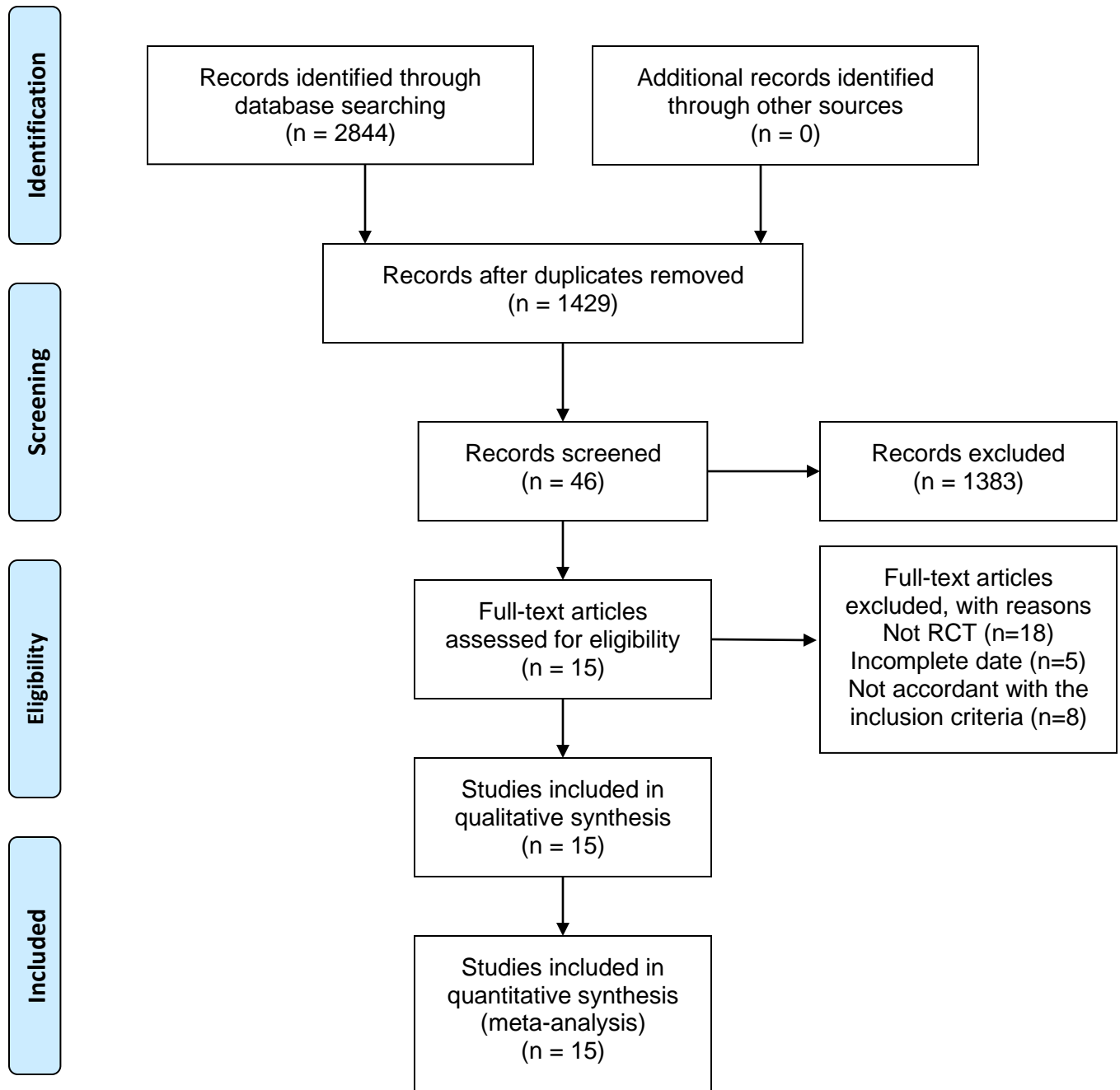

Supplement: S2 Checklist — (PDF) [file pone.0284411.s002.pdf]
