# Supplementary material for: Efficacy and safety of Weifuchun tablet for chronic atrophic gastritis: A systematic review and meta-analysis
Source: PLoS One. 2023 Apr 13;18(4):e0284411. doi: 10.1371/journal.pone.0284411 (PMC10101393; doi:10.1371/journal.pone.0284411)
Supplement: Attachment — Submitted filename: Response to Reviewers.docx [file pone.0284411.s003.docx]

Dear Prof. Mohamed Ezzat Abd El-Hack,

Thank you very much for giving us an opportunity to revise our manuscript! We appreciate editor and reviewers very much for their positive and constructive comments and suggestions on our manuscript entitled “Efficacy and safety of Weifuchun tablet for chronic atrophic gastritis: A systematic review and meta-analysis”(ID: PONE-D-22-32660), and we are submitting the revised manuscript for consideration for publication in PLOS ONE.

We have studied reviewer’s comments carefully and have made revision which marked in red in the paper. We have fully addressed the comments made by the reviewers and editor(see the attached “Rebuttal Letter” in this cover letter).

We would like to express our great appreciation to you and reviewers for comments on our paper. All authors have read and approved the re-submission of the manuscript! If you have any questions, please let me know!

Thank you for your consideration of our paper and we are looking forward to hearing from you!

Yours sincerely,

Longhua Wang

Department of gastroenterology,

Dongzhimen Hospital,

Beijing University of Chinese Medicine,

Beijing, China

Tel: 86-10-80816655-8107

E-mail: [wlh5698@126.com](mailto:wlh5698@126.com)

**Rebuttal Letter**

Point-by-point responses to editor and reviewers’ comments.

**Journal Requirements:**

1. Please ensure that your manuscript meets PLOS ONE's style requirements, including those for file naming. The PLOS ONE style templates can be found at

<https://journals.plos.org/plosone/s/file?id=wjVg/PLOSOne_formatting_sample_main_body.pdf> and <https://journals.plos.org/plosone/s/file?id=ba62/PLOSOne_formatting_sample_title_authors_affiliations.pdf>

**Response 1: Thanks for your reminding. We have downloaded the PLOS ONE style templates and fully addressed the format carefully.**

2. PLOS ONE does not copy edit accepted manuscripts ([https://journals.plos.org/plosone/s/criteria-for-publication#loc-5](https://journals.plos.org/plosone/s/criteria-for-publication%23loc-5)). To that effect, please ensure that your submission is free of typos and grammatical errors.

**Response 2: Thank you for reminding. We revised the whole manuscript carefully to avoid language errors. In addition, we consulted a professional editing service to check the English.**

3. We note that this manuscript is a systematic review or meta-analysis; our author guidelines therefore require that you use PRISMA guidance to help improve reporting quality of this type of study. Please upload copies of the completed PRISMA checklist as Supporting Information with a file name “PRISMA checklist”.

**Response 3: Thank you for your suggestion. We have uploaded an update PRISMA checklist as supporting information with a file according to the revised manuscript.**

4. Please include captions for your Supporting Information files at the end of your manuscript, and update any in-text citations to match accordingly. Please see our Supporting Information guidelines for more information: <http://journals.plos.org/plosone/s/supporting-information>

**Response 4: Thanks for your reminding. We have included captions for supporting information files at the end of our manuscript.**

5. In your Data Availability statement, you have not specified where the minimal data set underlying the results described in your manuscript can be found. PLOS defines a study's minimal data set as the underlying data used to reach the conclusions drawn in the manuscript and any additional data required to replicate the reported study findings in their entirety. All PLOS journals require that the minimal data set be made fully available. For more information about our data policy, please see <http://journals.plos.org/plosone/s/data-availability>.

Upon re-submitting your revised manuscript, please upload your study’s minimal underlying data set as either Supporting Information files or to a stable, public repository and include the relevant URLs, DOIs, or accession numbers within your revised cover letter. For a list of acceptable repositories, please see [http://journals.plos.org/plosone/s/data-availability#loc-recommended-repositories](http://journals.plos.org/plosone/s/data-availability%23loc-recommended-repositories). Any potentially identifying patient information must be fully anonymized.

Important: If there are ethical or legal restrictions to sharing your data publicly, please explain these restrictions in detail. Please see our guidelines for more information on what we consider unacceptable restrictions to publicly sharing data:[http://journals.plos.org/plosone/s/data-availability#loc-unacceptable-data-access-restrictions](http://journals.plos.org/plosone/s/data-availability%23loc-unacceptable-data-access-restrictions). Note that it is not acceptable for the authors to be the sole named individuals responsible for ensuring data access.

We will update your Data Availability statement to reflect the information you provide in your cover letter.

**Response 5:** **Thank you for your suggestion! We have uploaded our study’s dataset to Figshare.** [**https://doi.org/10.6084/m9.figshare.22298005.v2**](https://doi.org/10.6084/m9.figshare.22298005.v2)

6. Please review your reference list to ensure that it is complete and correct. If you have cited papers that have been retracted, please include the rationale for doing so in the manuscript text, or remove these references and replace them with relevant current references. Any changes to the reference list should be mentioned in the rebuttal letter that accompanies your revised manuscript. If you need to cite a retracted article, indicate the article’s retracted status in the References list and also include a citation and full reference for the retraction notice.

**Response 6: Thanks for your reminding. We have reviewed our reference list carefully to ensure that it is complete and correct. According to Reviewer #2 comments, we added a reference in the revised manuscript( Lines 464-467).**

**Reviewers' comments:**

**Reviewer #1**: The meta-analysis revealed that there had a significant statistic difference in effectiveness compared Weifuchun with control group, but there was no significant difference in safety. More high-quality clinical studies are needed in the future. nice work by the authors

**Response: Thanks for the reviewer’s positive comments.**

**Reviewer #2:** The valuable study was well-designed and conducted, and the authors reported methods and results clearly and appropriately. However, some minor concerns need to be addressed as follows:1) Abstract Line 30-31, what does “Chinese patent medicine + western medicine” mean? A more precise description is expected.2) Introduction section: the necessity of performing this study should be highlighted and elaborated. 3)Methods section: Lines 96, 104, 107, 117, and 125, the name of researchers should be provided; 3) Discussion section, the authors should interpret the results further, place them in the context of currently available findings, explain in-depth why does Weifuchun tablet work better for ACG? and 4) The manuscript needs extensive revision for language and grammar.

**Response: Thanks for the reviewer’s positive and constructive comments and suggestions. Point-by-point responses as follows:**

**Comment 1**: Abstract Line 30-31, what does “Chinese patent medicine + western medicine” mean? A more precise description is expected.

**Response 1:** **Thank you for your suggestion! We have changed the description to “Western medicine and other Chinese patent medicine” to avoid confusing.(Line 31)**

**Comment 2:** Introduction section: the necessity of performing this study should be highlighted and elaborated.

**Response 2: Thanks for your suggestion! We have highlighted the necessity of performing this study in introduction section( Line 70-71).**

**Comment 3:** Methods section: Lines 96, 104, 107, 117, and 125, the name of researchers should be provided.

**Response 3: Thanks for your advice! We have added the name of researchers in methods section.(Lines 82, 100, 108, 111, 121-122, 126, and 131)**

**Comment 4:** Discussion section, the authors should interpret the results further, place them in the context of currently available findings, explain in-depth why does Weifuchun tablet work better for ACG?

**Response 4: Thank you for your suggestion! We have further explained and elaborated why WFC was better for CAG in discussion section ( Lines 266-278).**

**Comment 5:** The manuscript needs extensive revision for language and grammar.

**Response 5: Thanks for your suggestion! We revised the whole manuscript carefully to avoid language errors. In addition, we consulted a professional editing service to check the English.**
